# A cell-type-specific dynamic Bayesian network model for spontaneous and optogenetically evoked activity in the primary visual cortex

**DOI:** 10.1186/1471-2202-12-S1-O5

**Published:** 2011-07-18

**Authors:** Ali Mohebi, Jessica A Cardin, Karim G Oweiss

**Affiliations:** 1 Department of Electrical and Computer Engineering, Michigan State University, East Lansing, MI 48824, USA; 2 Neuroscience Program, Michigan State University, East Lansing, MI 48824, USA,; 3 Department of Neurobiology, Yale University School of Medicine, New Haven, CT 06510, USA

## 

Reciprocal interaction between excitatory and inhibitory neurons within and between layers of the cerebral cortex is a major element of brain function. Ensemble extracellular recording techniques using mircroelectrode arrays have permitted recording spiking activity of many neurons simultaneously to characterize network function [1]. Identifying the type of neurons in these recordings is not straightforward due to the variability in extracellular spike shapes, and the irregularities often observed in their interspike interval characteristics. In this study, we used optogenetic tools to genetically target fast spiking interneurons in the primary visual cortex of mice [2]. We modulated their spiking activity by illuminating the region with very short pulses (<1 ms) of light (~470 nm wavelength)in mice primary visual cortex. Using Dynamic Bayesian Network analysis [3], we assessed the effective connectivity between the recorded neurons in the presence and absence of light stimuli under distinct cortical states observed under light anesthesia. DBNs could identify the effective connectivity between putative excitatory pyramidal cells and inhibitory interneurons. These findings suggest a novel and unprecedented way to identify cortical neuronal circuits and characterize the dynamics of their computations *in vivo*.
